# Two Novel Dermaseptin-Like Antimicrobial Peptides with Anticancer Activities from the Skin Secretion of *Pachymedusa dacnicolor*

**DOI:** 10.3390/toxins8050144

**Published:** 2016-05-12

**Authors:** Daning Shi, Xiaojuan Hou, Lei Wang, Yitian Gao, Di Wu, Xinping Xi, Mei Zhou, Hang Fai Kwok, Jinao Duan, Tianbao Chen, Chris Shaw

**Affiliations:** 1 Natural Drug Discovery Group, School of Pharmacy, Queen’s University, Belfast BT9 7BL, Northern Ireland, UK; dshi01@qub.ac.uk (D.S.); l.wang@qub.ac.uk (L.W.); ygao07@qub.ac.uk (Y.G.); dwu03@qub.ac.uk (D.W.); t.chen@qub.ac.uk (T.C.); chris.shaw@qub.ac.uk (C.S.); 2 Tumor Immunology and Gene Therapy Center, Eastern Hepatobiliary Surgery Hospital, The Second Military Medical University, Shanghai 200438, China; xiaojuanhou07@163.com; 3 Faculty of Health Sciences, University of Macau, Avenida de Universidade, Taipa, Macau SAR, China; 4 Jiangsu Key Laboratory for Traditional Chinese Medicine (TCM) Formulae Research, Nanjing University of Chinese Medicine, Nanjing 210046, China; dja@njutcm.edu.cn

**Keywords:** amphibian, peptide, antimicrobial, anticancer, molecular cloning

## Abstract

The dermaseptin antimicrobial peptide family contains members of 27–34 amino acids in length that have been predominantly isolated from the skins/skin secretions of phyllomedusine leaf frogs. By use of a degenerate primer in Rapid amplification of cDNA ends (RACE) PCR designed to a common conserved domain within the 5′-untranslated regions of previously-characterized dermaseptin encoding cDNAs, two novel members of this peptide family, named dermaseptin-PD-1 and dermaseptin-PD-2, were identified in the skin secretion of the phyllomedusine frog, *Pachymedusa dacnicolor*. The primary structures of both peptides were predicted from cloned cDNAs, as well as being confirmed by mass spectral analysis of crude skin secretion fractions resulted from reversed-phase high-performance liquid chromatography. Chemically-synthesized replicates of dermaseptin-PD-1 and dermaseptin-PD-2 were investigated for antimicrobial activity using standard model microorganisms (Gram-positive bacteria, Gram-negative bacteria and a yeast) and for cytotoxicity using mammalian red blood cells. The possibility of synergistic effects between the two peptides and their anti-cancer cell proliferation activities were assessed. The peptides exhibited moderate to high inhibition against the growth of the tested microorganisms and cancer cell lines with low haemolytic activity. Synergistic interaction between the two peptides in inhibiting the proliferation of *Escherichia coli* and human neuronal glioblastoma cell line, U251MG was also manifested.

## 1. Introduction

Amphibian skins contain a well-developed chemical defense system comprised of gene-encoded antimicrobial peptides and those with a wide range of pharmacological activities [1,2]. These peptides are synthesized and stored in the granular glands and are released when required, usually in response to a stressor, such as a predator attack or infection [3]. A large number of antimicrobial peptides have been studied and it has been shown that among different amphibian species, the expression of antimicrobial peptides is genetically determined and that they exhibit highly-conserved structures in their signal peptide sequences. However, each species secretes their own individual set of antimicrobial peptides, which may only differ in a few amino acid residues within their sequences [4,5]. These differences have been suggested to be due to the need to be effective against diverse microorganisms in their environments [6].

To date, a large number of amphibian skin antimicrobial peptides have been identified, some of which have demonstrated outstanding antimicrobial activities. For example, pexiganan, an analogue of magainin 2, which is one of the first antimicrobial peptides isolated from amphibian skins, has entered Phase III clinical trials for its antibacterial activity in the management of diabetic foot ulcers [7,8,9,10]. In addition, the bombinins, isolated from *Bombina* toad species, showed broad antimicrobial spectrum while maintaining virtually inactive in haemolysis [11,12]. Additionally, brevinins, abundant in several ranid frog species, demonstrated potent antibacterial and anticancer activities, owning to their unique *C*-terminal disulfide-bridged cyclic heptapeptide, namely “rana box” [13,14,15]. Moreover, the dermaseptins, isolated from the skin of South and Central American phyllomedusine leaf frogs, exhibited broad antimicrobial spectrum with greater sensitivity to gram-negative bacteria, and remarkably inhibitory effect against HIV infection *in vitro* [16,17,18].

The dermaseptin (DSs) family of peptides isolated from the skin secretions of phyllomedusine frogs have been deemed interesting, since the isolation of the prototype peptide of this family, dermaseptin s1, a 34 amino acid residue peptide from *Phyllomedusa sauvagii* [19], was found to have inhibitory effects on filamentous fungi which can cause lethal infections. After this, dermaseptin family peptides were also found to have activity against many other pathogenic microorganisms including bacteria, yeasts and protozoans, but to have little effects on mammalian cells at effective concentrations [16,20,21,22]. At present, more than 50 different dermaseptin peptides have been identified from phyllomedusine frog species [23]. These peptides are cationic (Lys-rich) molecules of 24–34 amino acids and have the ability to fold into amphipathic α-helical structures when being presented in hydrophobic media [16,24]. Within their primary structures, family members also share common signatures consisting of a conserved Trp residue at position 3 and a conserved sequence, −AA(A/G)KAAL(G/N)A−, in their mid-regions [23]. Interestingly, although there has been intensive study on the broad-spectrum antimicrobial activities of most dermaseptin peptides, the research on their anticancer activity has been limited, with only two members of this family—dermaseptin B2 and B3 having been reported to exhibit anti-proliferative activity against human prostate, mammary and lymphoma cancer cells [25].

Here we report the isolation and characterization of two novel dermaseptins—dermaseptin-PD-1 and dermaseptin-PD-2 from the skin secretion of *Pachymedusa dacnicolor*. The core aim of this study was to determine the spectrum of bioactivity of the two novel dermaseptins, including their antimicrobial activity, both individually and in combination, and to assess their possible effects on the growth of human cancer cell lines. Toward this goal, here we describe the cloning and sequencing of two full-length cDNAs, each of which encodes the precursor of a novel dermaseptin antimicrobial peptide. The cDNAs were cloned from a cDNA library constructed from the mRNAs isolated from the lyophilized skin secretion of *Pachymedusa dacnicolor*. Parallelly, dermaseptin-PD-1 and dermaseptin-PD-2 were identified in separate reversed phase HPLC fractions of the crude skin secretion and confirmed of their primary structures by electrospray ion-trap MS/MS. Both peptides were subsequently chemically-synthesized for antimicrobial and anti-cancer studies.

## 2. Results

### 2.1. Identification of Dermaseptin-PD-1 and Dermaseptin-PD-2 Precursor cDNAs from a Skin Secretion-Derived cDNA Library

**Figure 1:**
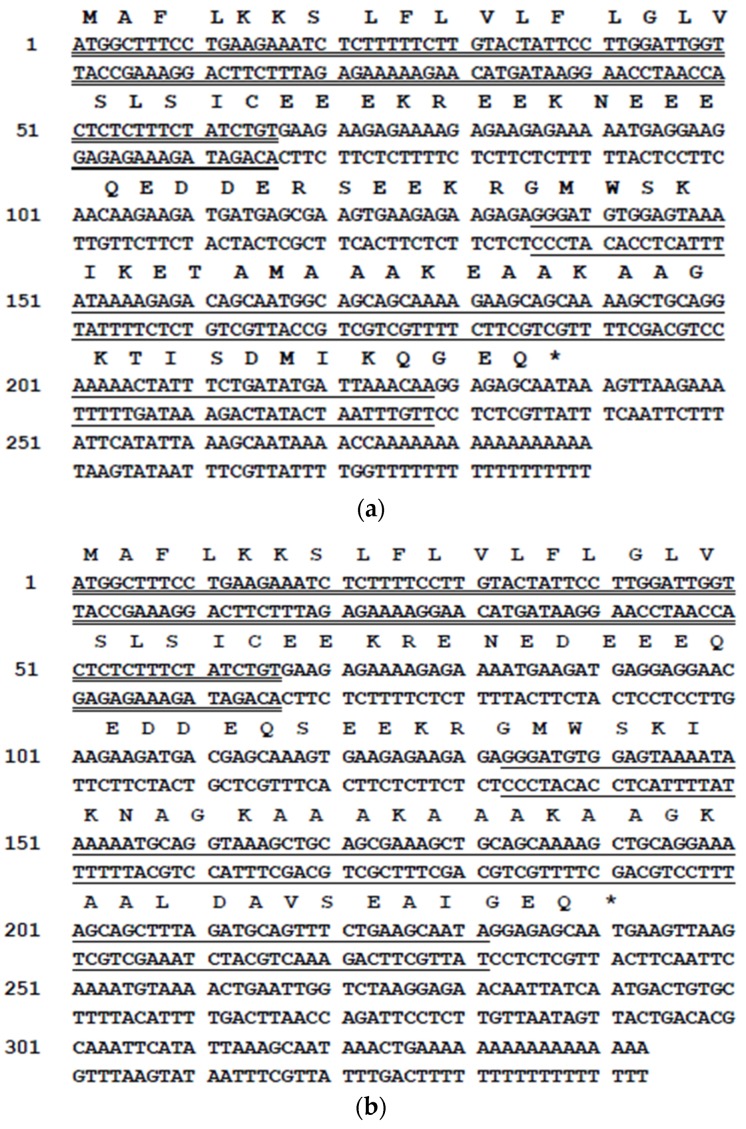
Nucleotide sequences of precursor cDNAs encoding dermaseptin-PD-1 (**a**) and dermaseptin-PD-2 (**b**) cloned from a *Pachymedusa dacnicolor* skin secretion library and deduced amino acid sequences of precursors. The nucleotide sequences of mature peptides are single-underlined, the nucleotide sequences of putative signal peptides are double-underlined and the stop codons are indicated by asterisks.

**Figure 2:**
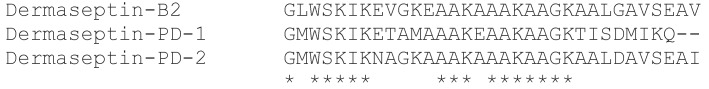
Alignment of amino acid sequences of dermaseptin-B2, dermaseptin-PD-1 and dermaseptin-PD-2. The identical amino acid residues are indicated by asterisks.

Two bands at approximately 300 bp and 400 bp were resolved in the gel electrophoresis following the Rapid amplification of cDNA ends (RACE) PCR (Figure S1). Cloning of the PCR product consistently produced two different cDNA sequences. The first cDNA encoded a precursor of a novel dermaseptin-related peptide named dermaseptin-PD-1 (Figure 1a) and its open reading frame consisted of 79 amino acid residues. The second cDNA contained an open-reading frame encoding a similar precursor to that of dermaseptin-PD-1 but consisting of 80 amino acids, including an identical signal peptide and a similar mature peptide, which was named dermaseptin-PD-2 (Figure 1b). Both predicted mature peptides had an identical *N*-terminal heptapeptide sequence—GMWSKIK—and an identical internal motif −AAAKE/AAAKAAGK−. Each mature peptide sequence was preceded by a Lys-Arg motif, which is a typical endoproteolytic cleavage site. Alignment of the amino acid sequence of the mature peptides of dermaseptin-B2, dermaseptin-PD-1 and dermaseptin-PD-2, which was performed using Clustal tool, revealed high degree of conservation in the sequence of the three demaseptin peptides and their internal motif (Figure 2).

### 2.2. Isolation and Structural Characterisation of Dermaseptin-PD-1 and Dermaseptin-PD-2

**Figure 3:**
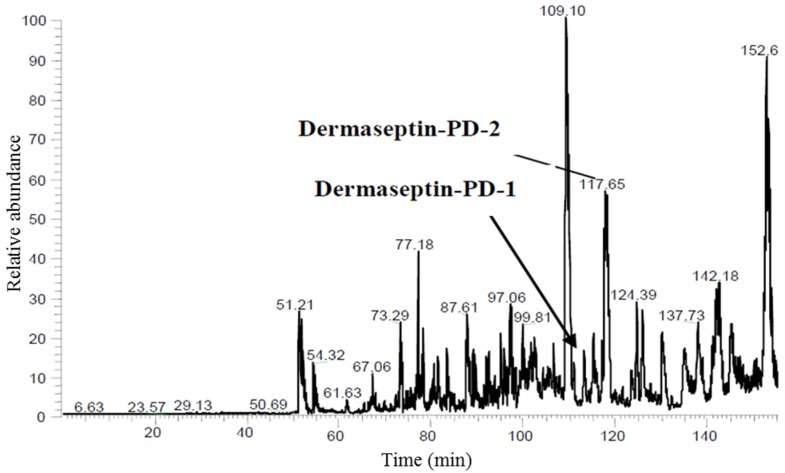
Reversed-phase HPLC chromatogram of *Pachymedusa dacnicolor* skin secretion indicating elution positions/retention times of the two novel dermaseptin peptides (arrows). *X*-axis indicates retention time in min and the *Y*-axis indicates relative absorbance at 214 nm in arbitrary units.

**Figure 4:**
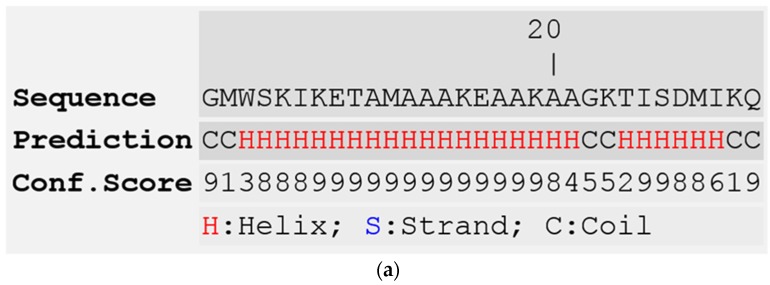

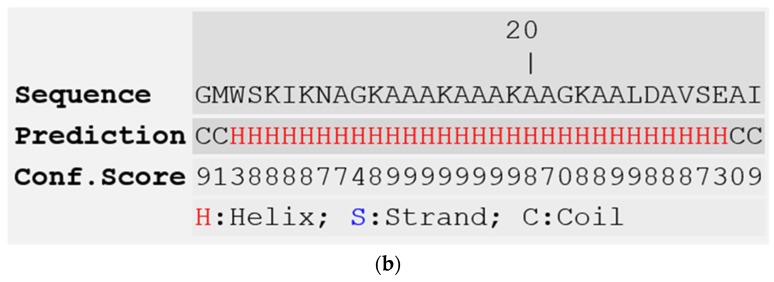
Predicted secondary structures of (**a**) dermaseptin-PD-1 and (**b**) dermaseptin-PD-2 using I-TASSER online server.

Reversed-phase HPLC of crude skin secretion was performed to attempt identification and structural confirmation of the putative novel dermaseptins encoded by the cloned cDNAs (Figure 3). To this end, a sample from each chromatographic fraction was subjected to MALDI-TOF mass spectrometric analysis. Once fractions containing peptides coincident in molecular masses to those predicted from cloned cDNAs were identified, samples of these were further subjected to MS/MS fragmentation sequencing. The presence and identity of both predicted peptides were confirmed. The amino acid sequences of both peptides, as determined by MS/MS sequencing, are shown in Table 1 and Table 2. The secondary structures of both peptides, predicted using I-TASSER online server [26], revealed that they contained a large proportion of α-helical region (Figure 4). Although the α-helical region of dermaseptin-PD-1 is interrupted by a short coil, which consists of two amino acid residues, dermaseptin-PD-1 exhibits continuous and intact α-helical region.

### 2.3. Solid Phase Synthesis of Dermaseptin-PD-1 and Dermaseptin-PD-2

After the unequivocal determination of the primary structures of dermaseptin-PD-1 and dermaseptin-PD-2, both peptides were successfully synthesized using a solid phase Fmoc chemical method. The synthetic peptides were then subjected to RP-HPLC to determine degree of purity and to MALDI-TOF MS and MS/MS fragmentation to establish authenticity of structures.

### 2.4. Antimicrobial and Cytotoxic Activities

**Figure 5:**
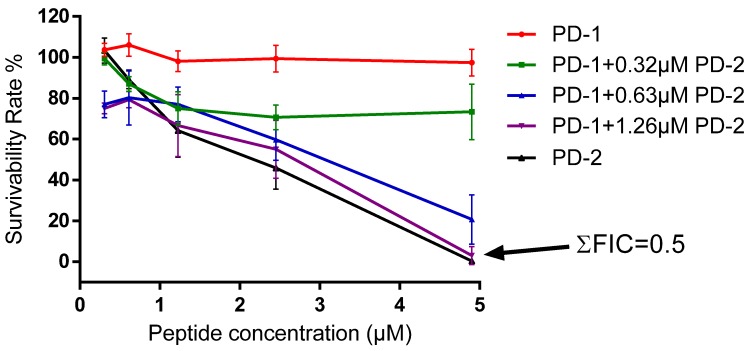
Synergistic effect between dermaseptin-PD-1 (PD-1) and dermaseptin-PD-2 (PD-2) against the growth of the Gram-negative bacterium, *E. coli*. The best combination concentrations of dermaseptin-PD-1 and dermaseptin-PD-2 are 4.9 μM and 1.26 μM, respectively. The ΣFIC was calculated as 0.5.

Dermaseptin-PD-1 exhibited a broad-spectrum of activity against all three tested microorganisms. However, it was found to be more effective against *E. coli* than against either *S. aureus* or *C. albicans*. The minimum inhibitory concentrations (MICs) and minimum bactericidal concentrations (MBCs) obtained with this peptide are shown in Table 3. Dermaseptin-PD-2, in contrast, had an identical antibacterial spectrum but exhibited a higher potency on all tested microorganisms (Table 3). The lethal concentration values of dermaseptin-PD-2 on tested microorganisms are also shown in Table 2. Both peptides did not possess significant hemolytic activity. The possible synergistic effects between dermaseptin-PD-1 and dermaseptin-PD-2 were checked using a checkerboard assay. The combination of both peptides showed a synergistic effect on the tested Gram-negative bacterium, *E. coli* at the concentrations of 4.9 μM and 1.26 μM, respectively. The calculated lowest cumulative fractional inhibitory concentration (ΣFIC), which is used to measure the combination effect of different compounds, is 0.5 (Figure 5).

### 2.5. Assessment of Activity of Peptides on Human Cancer Cell Lines

**Figure 6:**
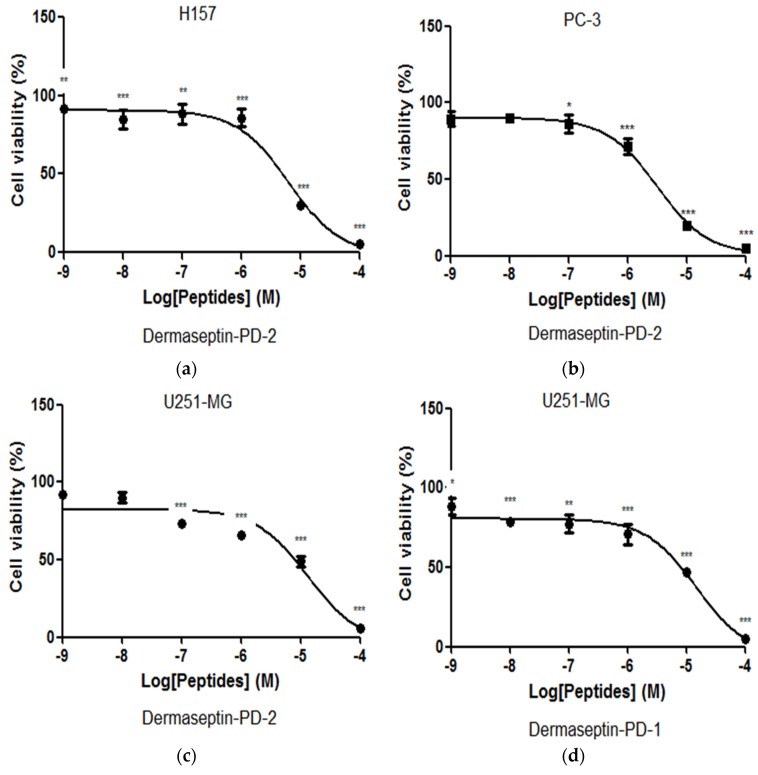

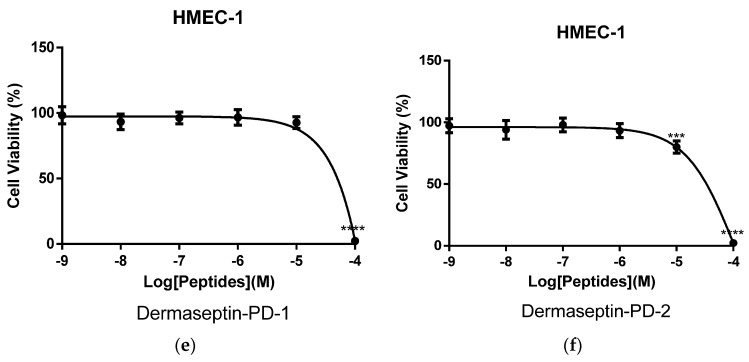
The effect on proliferation of cancer cell lines, H157 (**a**), PC-3 (**b**) and U251-MG (**c**) after treatment with dermaseptin-PD-2 within the concentration range of 10^−9^ to 10^−4^ M. The effect on proliferation of cancer cell line U251 MG (**d**) after treatment with dermaseptin-PD-1 within the concentration range of 10^−9^ to 10^−4^ M. The effect on normal human cell line, human microvessel endothelial cell, HMEC-1 after treatment with dermaseptin-PD-1 (**e**) and dermaseptin-PD-2 (**f**) within the concentration range of 10^−9^ to 10^−4^ M. * *p* < 0.05, ** *p* < 0.01, *** *p* < 0.001, **** *p* < 0.0001.

**Figure 7:**
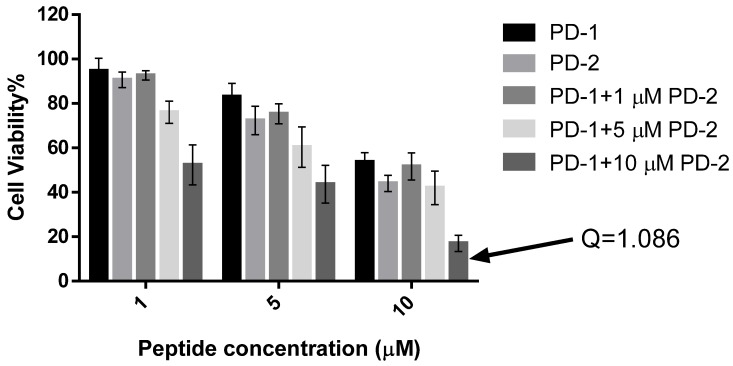
The effect on proliferation of human neuronal glioblastoma cell line, U251-MG after treatment with an array of combinations of dermaseptin-PD-1 (PD-1) and dermaseptin-PD-2 (PD-2). The arrow indicates the highest inhibitory rate of a combination of dermaseptin-PD-1 (10 μM) and dermaseptin-PD-2 (10 μM) and was used to calculate the combination index (*Q*), which is 1.086 (*Q* < 0.85, *Q* > 1.15 and 0.85 < *Q* < 1.15 indicate antagonism, synergy, and additive effect, respectively.)

Dermaseptin-PD-1 was found to have effects in modulating the growth of H157, PC-3 and U251 MG cancer cell lines at concentrations between 10^−4^ and 10^−9^ M (Figure 6a–c). The obvious growth inhibition of all cell lines observed with this peptide was evident above a concentration of 10^−6^ M. Dermaseptin-PD-2 had more potent effects on H157 and PC-3 cells than on U251 MG cells, with IC_50_ values 6.43 μM, 3.17 μM, and 13.43 μM, respectively. However, dermaseptin-PD-1 only had effect on the U251 MG cells with an IC_50_ value 15.08 μM and displayed no inhibition of the growth of PC-3 and H157 cells (Figure 6d). Meanwhile, both dermaseptins showed lower cytotoxicity effect on normal human cell line, HMEC-1 where the IC_50_ of dermaseptin-PD-1 and dermaseptin-PD-2 are 36.35 μM and 27.28 μM, respectively (Figure 6e,f). The synergism study of the combination of dermaseptin-PD-1 and dermaseptin-PD-2 on human cancer cells was performed using U251 MG cell line. However, the result only revealed an additive effect against the proliferation of U251 MG cells at the concentration of 10 μM for both dermaseptins (Figure 7) with a calculated combination index of 1.086.

## 3. Discussion

Here we report the isolation of two novel dermaseptin-like peptides from the skin secretion of *Pachymedusa dacnicolor*. Dermaseptin-PD-1 with 31 residues and dermaseptin-PD-2 with 33 residues, both display the characteristics of members of the dermaseptin family of amphibian skin antimicrobial peptides, including several lysine residues, a conserved tryptophan residue in the third position from the *N*-terminus of the mature peptide and a conserved amphipathic region in the center of the sequence [23]. The prepropeptides of dermaseptin-PD-1 and dermaseptin-PD-2 share identity in signal peptides and more than 80% identity in the sequences of the acidic spacer peptide regions when maximized for sequence alignment. In addition, both prepropeptides have the highly-conserved propeptide convertase processing sites found in other dermaseptin-family peptides [27].

The first isolation and biological evaluation of a dermaseptin peptide caused much interest, resulting in the isolation and study of a large number of dermaseptin-family peptides from members of the *Phyllomedusinae* subfamily. This was largely due to the fact that members of the dermaseptin family have been proven to have a broad spectrum of antimicrobial activity against bacteria, yeasts, protozoans and filamentous fungi [16,20,21,22]. In this report, the novel dermaseptin-like peptides described also displayed a broad-spectrum of activity against all microorganisms tested including a Gram-positive bacterium, a Gram-negative bacterium and a yeast. It has been found that dermaseptins can form amphipathic α-helical structures when they associate with lipid membranes and can thus rapidly destroy plasma membranes [24,28]. This rapid mode of action is unlikely to induce antibiotic-resistance. Additionally, it has been documented that dermaseptins have the capacity to differentiate between mammalian and microbial cell membranes [22], and many reports have stated that these peptides do not show significant activity against mammalian blood cells [21,22]. The data produced in this study in this respect also showed that these novel dermaseptins had no significant effects on horse red blood cells. These combined properties make this group of peptides promising candidates for future treatment of multidrug-resistant infections.

Many antimicrobial peptides display anticancer activity so that they draw much attention of researches on anticancer drug discovery [29]. Dermaseptins have been identified since 1990s with more than 50 analogues reported till now. In a recent study, two dermaseptin peptides, dermaseptin B2 and B3, have been reported to be effect against the proliferation of human prostate, mammary and lymphoma cancer cells [25]. The high degree of structural similarity between dermaseptin-PD-1, dermaseptin-PD-2 and dermaseptin B2 (Figure 2), especially that between the latter two peptides, may indicate similar anticancer effect of the two novel peptides, particularly on prostate cancer cells. Interestingly, synthetic dermaseptin-PD-2 not only inhibited the proliferation of PC3 cells, but also showed similar effect on human lung cancer cell line, H157 and neuronal glioblastoma cell line, U251MG. However, dermaseptin-PD-1 was different in potency and spectrum of activity, which was only effective on the proliferation of U251MG cells. In this study, dermaseptin-PD-2 showed a broader activity spectrum than dermaseptin-PD-1 on the human cancer cell lines and both of them exhibited low cytotoxicity on normal human cells, which makes dermaseptins more promising candidates in the discovery of new anticancer drugs.

Many previous studies have indicated that the mechanism of action of demaseptins on cancer cells is similar to their antimicrobial mechanisms, where electrostatic attraction plays important role and facilitates the interaction between these cationic peptides and the negatively-charged membranes of both microorganisms and cancer cells [29]. In this study, dermaseptin-PD-2 showed more potent antimicrobial activity and broader spectrum on the human cancer cell lines than dermaseptin-PD-1, which might be caused by the difference in their secondary structures. Dermaseptin-PD-2 contains one more positive charge than dermaseptin-PD-1 in the physiological environment, which could enhance the electrostatic attraction between the negatively-charged cell membrane and the peptides. Meanwhile, dermaseptin-PD-2 was predicted to form a continuous and larger α-helix region while the helix of dermaseptin-PD-1 was interrupted by a short coil (Figure 4). It is indicated that dermaseptin-PD-2 could exhibit more amphipathicity to enhance the effect on interaction between the peptide and cell membranes.

Dermaseptin-PD-1 and dermaseptin-PD-2 showed synergistic interaction on the tested Gram-negative bacterium, *E. coli*. However, there was only an additive effect of the combination of the two peptides on human cancer cells. Yet the mechanism behind has not been investigated, it could be explained by the natural skin defense system that is more responsible for preventing infection of microorganism instead of cancer cells. In addition, different peptides may have special targets in the interaction with complex cell wall and membrane of microorganisms. The synergy between different peptides on microorganism has also been found in other dermaseptins and other amphibian skin peptide families, such as the bombinins from bombinid toads [4,30].

In summary, the discovery of a large number of peptides in the skin secretions of amphibians with broad-spectrum antimicrobial bioactivity not only further highlights their role in the host defense system, but also suggest that they might be promising candidates for the discovery of anti-infectious agents. However, the modes of action of the peptides on both microorganism and cancer cells need to be investigated in the future.

## 4. Materials and Methods

### 4.1. Acquisition and Maintenance of Experimental Specimens

Adult specimens of *Pachymedusa dacnicolor* (*n* = 4) were obtained from a commercial source in the United States as small metamorphs and grown to adult size over 12 months. The frogs were maintained in a dedicated tropical frog facility, fed multivitamin-load crickets three times per week and at a temperature of 18–25 °C and 85% humidity, under a 12 h/12 h light/dark cycle. Sampling of skin secretion was performed by Mei Zhou under UK Animal (Scientific Procedures) Act 1986, project licence PPL 2694, issued by the Department of Health, Social Services and Public Safety, Northern Ireland. Procedures had been vetted by the IACUC of Queen's University Belfast, and approved on 1 March 2011.

### 4.2. Construction of a Skin Secretion-Derived cDNA Library and Cloning of Relevant cDNAs

Skin secretion was obtained by gently massaging the dorsal skin of the frogs. The secretion was washed from the skin using deionized water, snap-frozen in liquid nitrogen and lyophilized. Five milligrams of lyophilized skin secretion were dissolved in 1 mL of cell lysis/mRNA stabilization solution supplied in the kit (Life technologies, Oslo, Norway). The isolation of polyadenylated mRNA was achieved by using magnetic oligo-dT beads as described by the kit manufacturer. Afterwards, 3′RACE procedures were carried using a SMART-RACE kit (Clontech, Palo Alto, CA, USA) to achieve full-length prepropeptide nucleic acid sequence data. Briefly, the isolated mRNA was subjected to reverse transcription using a primer and reverse transcriptase (supplied by the kit manufacturer) to obtain full-length first-strand cDNA and then 3′-RACE procedures were performed using a UPM primer supplied in the kit and a degenerate sense primer (S1; 5′-ACTTTCYGAWTTRYAAGMCCAAABATG-3′) that was designed to a conserved region of the 5′-untranslated region of phylloxin cDNA from *Phyllomedusa bicolor* (EMBL Accession no. AJ251876) and the opioid peptide cDNA from *Pachymedusa dacnicolor* (EMBL Accession no. AJ005443). The PCR procedure was carried out under the following conditions: Initial denaturation: 90 s at 94 °C; annealing: 30 s at 58 °C; extension: 180 s at 72 °C—for 35 cycles. PCR products were analyzed by agarose gel electrophoresis and cloned into a pGEM-T vector system (Promega Corporation, Southampton, UK). Sequencing reactions were performed using a BigDye Terminator sequencing kit (Applied Biosystems, Foster City, CA, USA) and analyzed by an automated ABI 3100 DNA sequencer.

### 4.3. Reverse Phase HPLC Fractionation of Crude Skin Secretion and Amino-Acid Sequence Analysis of Relevant Peptides

A further five milligrams of lyophilized skin secretion were dissolved in 0.5 mL of 0.05% (*v*/*v*) trifluoroacetic acid (TFA)/water and clarified by centrifugation. The clear supernatant was subjected to reverse-phase HPLC on an analytical column (Jupiter C-5, 250 mm × 10 mm, Phenomenex, Macclesfield, Cheshire, UK), eluted with a 0%–80% linear gradient of acetonitrile containing 0.05% (*v*/*v*) TFA in 240 min at a flow rate of 1 mL/min. Absorbance was monitored at 214 nm. Fractions were collected at minute intervals and samples from each were analyzed by matrix-assisted laser desorption/ionization, time-of-flight mass spectrometry (MALDI-TOF MS) (Perseptive Biosystems, Voyager DE, Perseptive Biosystems, Foster City, CA, USA). The fraction(s) containing peptides with molecular masse coincident with those calculated from cloned precursor-encoding cDNAs were then subjected to MS/MS fragmentation sequencing using an electrospray ion-trap mass spectrometer (Thermo Fisher Scientific, San Francisco, CA, USA).

### 4.4. Solid-Phase Peptide Synthesis

Once the primary structures of the novel peptides had been determined unequivocally, they were separately synthesized by solid-phase Fmoc chemistry using a PS3 automated synthesizer (Protein Technologies, Inc., Tucson, AZ, USA). Synthesis was performed using an HBTU activator, dimethylformamide (DMF), *N*-methylmorpholine (NMM) and piperidine. Cleavage of the peptides from the resin and side-chain deprotection were performed in a mixture composed of 95% TFA, 2.5% Triisopropylsilane (TIPS), 2.5% water (*v*/*v*/*v*) for 6 h at room temperature. Once the peptides were separated from the resin, they were precipitated using diethyl ether at −20 °C, washed three times, dissolved in 5 mL of 0.05% TFA/water and lyophilized. After lyophilization, the synthetic peptides were analyzed by reverse phase HPLC and MALDI-TOF mass spectrometry for determination of purity and authenticity of structure.

### 4.5. Antimicrobial Assays

The minimal inhibitory concentrations of synthetic peptides were determined using the Gram-positive bacterium, *Staphylococcus aureus* (NCTC 10788), the Gram-negative bacterium, *Escherichia coli* (NCTC 10418) and *Pseudomonas aeruginosa* (ATCC 27853), and the yeast, *Candida albicans* (NCPF 1467). These four microorganisms were grown in Mueller-Hinton Broth (Oxoid, Basingstoke, Hampshire, UK) (MHB) at 37 °C and optical densities (ODs) of culture media at λ550 nm were determined to assess dilution factors required to achieve inoculation concentrations of 5 × 10^5^ colony forming units (cfu)/mL. The highest peptide concentrations of 512 mg/L were prepared by dissolving peptides in DMSO and dilution series were then constructed by double-dilution in culture medium. The minimal inhibitory concentrations (MICs) were determined in 96-well microtitre plates. After the determination of the MICs of the peptides, 10 μL of medium from each well was inoculated onto Mueller Hinton agar (Oxoid, Basingstoke, Hampshire, UK) (MHA) plates. The minimal bactericidal concentrations (MBC) were determined as the lowest peptide concentrations from which no colonies were grown on the MHA plates.

### 4.6. Haemolysis Assay

The ability of peptides to lyse mammalian red blood cells was assessed using a 4% suspension of horse red blood cells (supplied by TCS Biosciences Ltd, Botolph Claydon, Buckingham, UK) in phosphate-buffered saline (PBS). The red blood cells were washed using phosphate-buffered saline (PBS) and centrifugation, which were repeated until the supernatant was clear. A series of peptide concentrations (512 mg/L–1 mg/L) were prepared in PBS. Five replicates of each peptide concentration were incubated with red blood cell suspension at 37 °C for 2 h. After 2 h, each cell-peptide mixture was centrifuged and 200 μL of each supernatant were added into wells of a 96-well plate. Lysis of red blood cells was assessed by measurement of supernatant optical densities using an ELISA plate reader (Biolise BioTek EL808, Winooski, VT, USA) set to λ550 nm. Negative controls were prepared using red cell suspension and PBS in equal volumes. Positive controls were prepared using red cell suspension and an equal volume of PBS containing 2% of the non-ionic detergent, Triton X-100 (Sigma-Aldrich, St. Louis, MO, USA).

### 4.7. Assessment of Effects of Peptides on Human Cancer Cell Lines Using the MTT Assay

Synthetic peptides were screened for cytotoxicity on the human prostate carcinoma cell line, PC-3 (ATCC-CRL-1435), the human non-small cell lung cancer cell line, H157 (ATCC-CRL-5802), the human breast cancer cell line MDA-MB-435s (ATCC-HTB-129), the human neuronal glioblastoma cell line U251MG (ECACC-09063001) and the human breast cancer non-tumorigenic mammary gland cell line, MCF-7 (ATCC-HTB-22). The PC-3 and H157 cell lines were cultured in RPMI-1640 culture medium (Invitrogen, Paisley, UK) with 10% added fetal bovine serum (FBS) (Sigma-Aldrich, St. Louis, MO, USA) and 1% Penicillin-Streptomycin (Invitrogen, Paisley, UK), and MDA-MB-435s, U251 MG, MCF-7 cell lines were cultured in Dulbecco’s Modified Eagle’s Medium (DMEM) (Invitrogen, Paisley, UK) with 10% added FBS and 1% Penicillin-Streptomycin (Invitrogen, Paisley, UK). Meanwhile, the cytotoxicity of synthetic peptides on normal human cell line was evaluated using human microvessel endothelial cell, HMEC-1 (ATCC-CRL-3243), and the cells were cultured in MCDB131 medium (Gibco, Paisley, UK) containing 10% FBS, 10 ng/mL EGF, 10 mM l-Glutamine and 1% penicillin streptomycin. The cell lines, PC-3, H157, MDA-MB-435s and HMEC-1, were supplied by American Type Culture Collection (ATCC, Teddington, Middlesex, UK) and U251MG was supplied by European Collection of Authenticated Cell Cultures (ECACC, Porton Down, Salisbury, UK). When the cells reached 80%–90% confluence, they were counted and seeded onto 96-well plates as 5000 cells in serum medium per well. Following a 24-h incubation, the cells were treated with serum-free medium. After a further 12 h, the cells were treated with a series of peptide concentrations (10^−9^–10^−4^ M) in serum-free medium, with each concentration and controls in 7 replicates. The plates were incubated for a further 24 h. After this, 10 μL of MTT solution were added into each well and after a 4-h incubation, the liquid in each well was removed and 100 μL of DMSO were added into each. The absorbance of each well was then measured by an EL808 TM Absorbance Microplate Reader (Biolise BioTek EL808, Winooski, VT, USA) set at 570 nm.

### 4.8. Assessment of Possible Synergism between the Two Peptides

The method of checkerboard titration was used to assess the possible synergistic interactions between the peptides on both antimicrobial and anticancer activities.

According to the MIC data obtained for each peptide, peptides were prepared between concentrations of 4 × MIC and 1 × MIC along the rows of a 96-well plate. The tested microorganism was *Escherichia coli* (NCTC 10418). Microorganism culture at 10^6^ cfu/mL, was inoculated into the 96-well plate. The plate was incubated for 18 h at 37 °C under humid conditions. After 18 h, optical densities of the bacterial cultures were measured using an ELISA plate reader (Biolise BioTek EL808, Winooski, VT, USA). The lowest cumulative fractional inhibitory concentration (ΣFIC) could then be determined using the following formula to assess the degrees of synergy:

ΣFIC = A/MIC_A_ + B/MIC_B_

where A and B represent the respective MICs when both peptides were in combination and MIC_A_ and MIC_B_ represent the MICs for individual peptide A and peptide B. When ΣFIC ≤ 0.5, it indicates synergy, whereas when 0.5 < Σ FIC ≤ 1, it indicates an additive effect [31,32].

For assessing the synergetic activity of two dermaseptins against the growth of cancer cell lines, the method was modified from the previous study [33]. The tested cancer cell line was the human neuronal glioblastoma cell line, U251MG. It was cultured and prepared as Section 4.8. After treated with serum-free medium, three concentrations of both dermaseptins, 10 μM, 5 μM and 1 μM, were used to set a combination array along the rows of a 96-well plate and the plate was subsequent put into an incubator for 24 h. Then, MTT solution was added into wells followed by 4 h incubation, the formazan crystals was dissolved by adding 100 μL of DMSO. The synergism effect of two dermaseptins was calculated as the formula below:

*Q* = E_a+b_/(E_a_ + E_b_ − E_a_ × E_b_)

where *Q* is the combination index; E_a+b_ represents the cell proliferative inhibition rate of two dermaseptins; E_a_ and E_b_ represents the cell proliferative inhibition rate for individual peptide A and peptide B. When *Q* > 1.15, it indicates synergy, whereas when 0.85 < *Q* < 1.15, it indicates an additive effect [33].

## Figures and Tables

**Table 1:** Predicted b-ion and y-ion MS/MS fragment ion series (singly- and doubly-charged) of dermaseptin-PD-1. The actual ions detected in MS/MS spectra are colored red and blue.

#1	b(1+)	b(2+)	Sequence	y(1+)	y(2+)	#2
1	58.0288	29.518	G	-	-	31
2	189.0693	95.0383	M	3207.7053	1604.3563	30
3	375.1486	188.0779	W	3076.6648	1538.836	29
4	462.1806	231.5939	S	2890.5855	1445.7964	28
5	590.2756	295.6414	K	2803.5534	1402.2804	27
6	703.3596	352.1835	I	2675.4584	1338.2329	26
7	831.4546	416.2309	K	2562.3744	1281.6908	25
8	960.4972	480.7522	E	2434.2794	1217.6433	24
9	1061.5449	531.2761	T	2305.2368	1153.122	23
10	1132.582	566.7946	A	2204.1891	1102.5982	22
11	1263.6225	632.3149	M	2133.152	1067.0796	21
12	1334.6596	667.8335	A	2002.1115	1001.5594	20
13	1405.6968	703.352	A	1931.0744	966.0408	19
14	1476.7339	738.8706	A	1860.0373	930.5223	18
15	1604.8288	802.9181	K	1789.0002	895.0037	17
16	1733.8714	867.4394	E	1660.9052	830.9562	16
17	1804.9086	902.9579	A	1531.8626	766.4349	15
18	1875.9457	938.4765	A	1460.8255	730.9164	14
19	2004.0407	1002.524	K	1389.7883	695.3978	13
20	2075.0778	1038.0425	A	1261.6934	631.3503	12
21	2146.1149	1073.5611	A	1190.6563	595.8318	11
22	2203.1364	1102.0718	G	1119.6191	560.3132	10
23	2331.2313	1166.1193	K	1062.5977	531.8025	9
24	2432.279	1216.6431	T	934.5027	467.755	8
25	2545.3631	1273.1852	I	833.455	417.2311	7
26	2632.3951	1316.7012	S	720.3709	360.6891	6
27	2747.4221	1374.2147	D	633.3389	317.1731	5
28	2878.4626	1439.7349	M	518.312	259.6596	4
29	2991.5466	1496.277	I	387.2715	194.1394	3
30	3119.6416	1560.3244	K	274.1874	137.5973	2
31	-	-	Q-Amidated	146.0924	73.5499	1

**Table 2:** Predicted b-ion and y-ion MS/MS fragment ion series (singly- and doubly-charged) of dermaseptin-PD-2. The actual ions detected in MS/MS spectra are colored red and blue.

#1	b(1+)	b(2+)	Sequence	y(1+)	y(2+)	#2
1	58.0288	29.518	G	-	-	33
2	189.0693	95.0383	M	3111.7462	1556.3767	32
3	375.1486	188.0779	W	2980.7057	1490.8565	31
4	462.1806	231.5939	S	2794.6263	1397.8168	30
5	590.2756	295.6414	K	2707.5943	1354.3088	29
6	703.3596	352.1835	I	2579.4993	1290.2533	28
7	831.4546	416.2309	K	2466.4153	1233.7113	27
8	945.4975	473.2524	N	2338.3203	1169.6638	26
9	1016.5347	508.771	A	2224.2774	1112.6423	25
10	1073.5561	537.2817	G	2153.2402	1077.1238	24
11	1201.6511	601.3292	K	2096.2188	1048.613	23
12	1272.6882	636.8478	A	1968.1238	984.5655	22
13	1343.7253	672.3663	A	1897.0867	949.047	21
14	1414.7625	707.8849	A	1826.0496	913.5284	20
15	1542.8574	771.9324	K	1755.0124	878.0099	19
16	1613.8946	807.4509	A	1626.9175	813.9624	18
17	1684.9317	842.9695	A	1555.8804	778.4438	17
18	1755.9688	878.488	A	1484.8432	742.9253	16
19	1884.0638	942.5355	K	1413.8061	707.4067	15
20	1955.1009	978.0541	A	1285.7111	643.3592	14
21	2026.138	1013.5726	A	1214.674	607.8407	13
22	2083.1595	1042.0834	G	1143.6369	572.3221	12
23	2211.2544	1106.1309	K	1086.6154	543.8114	11
24	2282.2916	1141.6494	A	958.5205	479.7639	10
25	2353.3287	1177.168	A	887.4833	444.2453	9
26	2466.4128	1233.71	L	816.4462	408.7268	8
27	2581.4397	1291.2235	D	703.3622	352.1847	7
28	2652.4768	1326.7421	A	588.3352	294.6712	6
29	2751.5452	1376.2763	V	517.2981	259.1527	5
30	2838.5773	1419.7923	S	418.2297	209.6185	4
31	2967.6199	1484.3136	E	331.1976	166.1025	3
32	3038.657	1519.8321	A	202.155	101.5812	2
33	-	-	I-Amidated	131.1179	66.0626	1

**Table 3:** Minimum inhibitory concentrations (MICs) and minimum bactericidal concentrations (MBCs) of the two novel peptides (dermaseptin-PD-1 and dermaseptin-PD-2). Hemolytic effect was determined in comparison to 100% lysis of the red blood cells effected by incubation with 2% Triton X-100 for 2 h.

Peptides	MIC (μM)				MBC (μM)				Hemolysis (μM)
	*E. coli*	*S. aureus*	*C. albicans*	*P. aeruginosa*	*E. coli*	*S. aureus*	*C. albicans*	*P. aeruginosa*	Horse Red Cells
**Dermaseptin-PD-1**	19.6	39.2	39.2	19.6	39.2	78.4	78.4	78.4	>156.8
**Dermaseptin-PD-2**	5.0	5.0	10.1	2.5	20.2	10.1	20.2	10.1	>161.6

## Supplementary Materials

The following are available online at www.mdpi.com/2072-6651/8/5/144/s1. Figure S1: Gel electrophoresis of RACE PCR products from *Pachymedusa dacnicolor* skin secretion cDNAs library.

## Data Deposition Footnote

The nucleotide sequences of clones encoding dermaseptin-PD-1 and dermaseptin-PD-2, from the skin secretion of *Pachymedusa dacnicolor*, have been deposited in the EMBL Nucleotide Sequence Database under the accession codes, LN890478 and LN890479, respectively.

## Abbreviations

The following abbreviations are used in this manuscript:

MDPI	Multidisciplinary Digital Publishing Institute
DOAJ	Directory of open access journals
HPLC	High performance liquid chromatography
cDNA	complmentary deoxyribonucleic acid
BLAST	Basic Local Alignment Search Tool
NCBI	National Centre for Biotechnological Information
MALDI-TOF	Matrix-assisted laser desorption/ionisation, time-of-flight
MS	mass spectrometry
MS/MS	Tandem mass spectrometry
SEM	Standard error of the mean
TFA	Trifluoroacetic acid
PS	Peptide synthesiser
LC/MS/MS	Liquid chromatography coupled tandem mass spectrometry
