# Supplementary material for: Two Novel Dermaseptin-Like Antimicrobial Peptides with Anticancer Activities from the Skin Secretion of Pachymedusa dacnicolor
Source: Toxins (Basel). 2016 May 12;8(5):144. doi: 10.3390/toxins8050144 (PMC4885059; doi:10.3390/toxins8050144)
Supplement: Supplementary file 1 [file toxins-08-00144-s001.pdf]

Supplementary Materials: Two Novel Dermaseptin-Like Antimicrobial Peptides with Anticancer Activities from the Skin Secretion of *Pachymedusa dacnicolor*

Daning Shi, Xiaojuan Hou, Lei Wang, Yitian Gao, Di Wu, Xinping Xi, Mei Zhou, Hang Fai Kwok, Jinao Duan, Tianbao Chen and Chris Shaw

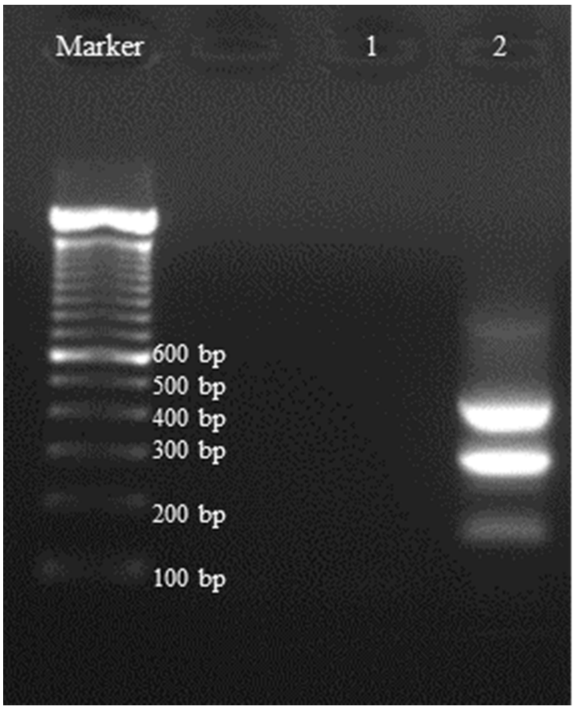

Figure S1. Gel electrophoresis of RACE PCR products from *Pachymedusa dacnicolor* skin secretion cDNAs library. The negative control (1) was performed using PCR grade water instead of the cDNA template which was used in the sample (2).
